# Validity of Estimating the Maximal Oxygen Consumption by Consumer Wearables: A Systematic Review with Meta-analysis and Expert Statement of the INTERLIVE Network

**DOI:** 10.1007/s40279-021-01639-y

**Published:** 2022-01-24

**Authors:** Pablo Molina-Garcia, Hannah L. Notbohm, Moritz Schumann, Rob Argent, Megan Hetherington-Rauth, Julie Stang, Wilhelm Bloch, Sulin Cheng, Ulf Ekelund, Luis B. Sardinha, Brian Caulfield, Jan Christian Brønd, Anders Grøntved, Francisco B. Ortega

**Affiliations:** 1 PROFITH (PROmoting FITness and Health Through Physical Activity) Research Group, Department of Physical Education and Sports, Faculty of Sport Sciences, University of Granada, Carretera de Alfacar s/n, 18071 Granada, Spain; 2 Physical Medicine and Rehabilitation Service, Biohealth Research Institute, Virgen de Las Nieves University Hospital, Jaén Street, s/n, 18013 Granada, Spain; 3 Institute of Cardiovascular Research and Sports Medicine, Department of Molecular and Cellular Sports Medicine, German Sport University, Cologne, Germany; 4 Department of Physical Education, Exercise Translational Medicine Centre, The Key Laboratory of Systems Biomedicine, Ministry of Education, and Exercise, Health and Technology Centre, Shanghai Jiao Tong University, Shanghai, China; 5 Insight Centre for Data Analytics, University College Dublin, Dublin, Ireland; 6 School of Public Health, Physiotherapy and Sport Science, University College Dublin, Dublin, Ireland; 7 School of Pharmacy and Biomolecular Sciences, Royal College of Surgeons in Ireland, Dublin, Ireland; 8 Exercise and Health Laboratory, CIPER, Faculdade de Motricidade Humana, Universida de de Lisboa, Lisbon, Portugal; 9 Department of Sport Medicine, Norwegian School of Sport Sciences, Oslo, Norway; 10 Department of Sports Science and Clinical Biomechanics, Research Unit for Exercise Epidemiology, Centre of Research in Childhood Health, University of Southern Denmark, Odense M, Denmark; 11 Faculty of Sport and Health Sciences, University of Jyväskylä, Jyvaskyla, Finland; 12 Department of Bioscience and Nutrition, Karolinska Institutet, Huddinge, Sweden

## Abstract

**Background:**

Technological advances have recently made possible the estimation of maximal oxygen consumption (*V*O_2max_) by consumer wearables. However, the validity of such estimations has not been systematically summarized using meta-analytic methods and there are no standards guiding the validation protocols.

**Objective:**

The aim was to (1) quantitatively summarize previous studies investigating the validity of the *V*O_2max_ estimated by consumer wearables and (2) provide best-practice recommendations for future validation studies.

**Methods:**

First, we conducted a systematic review and meta-analysis of studies validating the estimation of *V*O_2max_ by wearables. Second, based on the state of knowledge (derived from the systematic review) combined with the expert discussion between the members of the Towards Intelligent Health and Well-Being Network of Physical Activity Assessment (INTERLIVE) consortium, we provided a set of best-practice recommendations for validation protocols.

**Results:**

Fourteen validation studies were included in the systematic review and meta-analysis. Meta-analysis results revealed that wearables using resting condition information in their algorithms significantly overestimated *V*O_2max_ (bias 2.17 ml·kg^−1^·min^−1^; limits of agreement − 13.07 to 17.41 ml·kg^−1^·min^−1^), while devices using exercise-based information in their algorithms showed a lower systematic and random error (bias − 0.09 ml·kg^−1^·min^−1^; limits of agreement − 9.92 to 9.74 ml·kg^−1^·min^−1^). The INTERLIVE consortium proposed six key domains to be considered for validating wearable devices estimating *V*O_2max_, concerning the following: the target population, reference standard, index measure, testing conditions, data processing, and statistical analysis.

**Conclusions:**

Our meta-analysis suggests that the estimations of *V*O_2max_ by wearables that use exercise-based algorithms provide higher accuracy than those based on resting conditions. The exercise-based estimation seems to be optimal for measuring *V*O_2max_ at the population level, yet the estimation error at the individual level is large, and, therefore, for sport/clinical purposes these methods still need improvement. The INTERLIVE network hereby provides best-practice recommendations to be used in future protocols to move towards a more accurate, transparent and comparable validation of *V*O_2max_ derived from wearables.

**PROSPERO ID:**

CRD42021246192.

**Supplementary Information:**

The online version contains supplementary material available at https://doi.org/10.1007/s40279-021-01639-y.

## Key Points

**Table:** 

Wearables using exercise-based algorithms provide higher accuracy in the estimation of maximal oxygen consumption (*V*O_2max_) than those based on resting conditions.
Wearables using exercise-based estimation seem to be optimal for measuring *V*O_2max_ at the population level, yet the estimation error at the individual level still needs further improvement.
In this article, the Towards Intelligent Health and Well-Being Network of Physical Activity Assessment (INTERLIVE) network provides best-practice recommendations to be used in future protocols to move towards a more accurate, transparent and comparable validation of *V*O_2max_ derived from wearables.

## Introduction

The use and development of wearable technology monitoring fitness and activity have grown exponentially over the last few years. In 2020, 396 million wearable units were shipped worldwide, and it is forecasted that this will increase up to 631.7 million units by 2024 [1]. Wearable devices give users the opportunity to monitor health-related metrics, such as daily steps, heart rate (HR), energy expenditure, or cardiorespiratory fitness, therefore, promoting physical activity and optimizing health and sports performance [2, 3]. Furthermore, the omnipresence of wearables enhances digital phenotyping at a population level, which offers valuable information about physical activity and fitness levels from around the world that can be used to guide global health promotion actions [2, 4].

The most accepted measure of cardiorespiratory fitness is maximal oxygen consumption (*V*O_2max_), which has been shown to be a powerful marker of health and has recently been proposed as a clinical vital sign by the American Heart Association [5]. Furthermore, *V*O_2max_ is widely known as a key indicator of endurance performance and, therefore, its measurement is of vital importance for sports performance in general [6]. The current guidelines for accurate testing of *V*O_2max_ require measurement of gas exchange by indirect calorimetry usually in a laboratory during an exercise test to exhaustion [7]. These tests require expensive equipment (e.g., gas analyzer) and trained technicians to collect and interpret the data, which makes *V*O_2max_ assessments less feasible for risk prediction in clinical practice and unaffordable for most recreational athletes and for the general population. Indirect estimation of *V*O_2max_ by submaximal field tests overcomes some of these disadvantages and offers acceptable estimations of *V*O_2max_ [8, 9]. However, the abovementioned digital era of consumer wearable devices opens new horizons for fitness monitoring without the need for laboratory or field testing.

In view of the enormous potential of these devices, wearable companies are making significant investments in research and development to provide valid fitness and activity measures, such as *V*O_2max_ [10, 11]. Previous systematic reviews have already assessed how well wearable devices estimate most of the health measures such as step count [12, 13], HR [14, 15], and energy expenditure [14, 16]; however, to the best of our knowledge, no systematic review or meta-analysis focusing on the validity of the estimated *V*O_2max_ is available. Furthermore, the current science behind the validation protocols of wearable devices suffers major limitations, mainly due to a lack of consensus and guidelines ensuring good practices [17, 18]. This is precisely one of the main goals of the Towards Intelligent Health and Well-Being Network of Physical Activity Assessment (INTERLIVE) consortium, which is to develop best-practice protocols for the validation of consumer wearable fitness and activity measures. The INTERLIVE consortium has already published guidelines adapted to the nature of specific fitness/physical activity measures such as step count [19] and HR [20]. However, to date there are no specific standards guiding both manufacturers and the scientific community in the validation of estimating *V*O_2max_ by consumer wearables.

Therefore, in this article, INTERLIVE had two main objectives: (1) to systematically summarize previous studies investigating the validity of *V*O_2max_ as estimated by consumer wearable devices based on a meta-analysis, and (2) to provide best-practice validation recommendations based on the systematic review of the literature together with an evidence-informed INTERLIVE consortium discussion.

## Methods: Expert Statement Process and Meta-Analysis

### The INTERLIVE Network

INTERLIVE (https://www.interlive.org/) is a consortium composed of six universities—University of Lisbon (Portugal), German Sport University (Germany), University of Southern Denmark (Denmark), Norwegian School of Sport Sciences (Norway), University College Dublin (Ireland), and University of Granada (Spain)—and one technology company, Huawei Technologies (Finland). The consortium was founded in 2019 and strives towards developing best-practice protocols for evaluating the validity of consumer wearables with regard to the measurement of exercise/activity metrics. Moreover, INTERLIVE aims to increase awareness of the advantages and limitations of different validation methods and to introduce novel health and performance-related metrics, fostering a widespread use of physical activity indicators.

### Expert Validation Process

The consortium followed the same process as was used previously [19, 20]. First, we conducted a systematic review of the scientific literature on the studies validating *V*O_2max_ estimated by consumer wearables against a reference standard (criterion measure). Second, the information obtained from the systematic review, together with previous related statements [17–21], was critically discussed within the consortium to provide guidelines and recommendations on how to conduct optimal validation protocols. Third, a set of key domains for best-practice recommendations was proposed based on the evidence-informed expert opinion of the INTERLIVE members.

### Systematic Review and Meta-Analysis Process

This systematic review was guided by the Preferred Reporting Items for Systematic Reviews and Meta-Analyses diagnostic test accuracy guideline. The protocol was registered in advance in the PROSPERO database (ID: CRD42021246192).

#### Data Sources and Search Strategy

PubMed, Web of Sciences, and Scopus databases were searched dating up to January 14, 2021. Members from the INTERLIVE network defined the search strategy, which can be found for replication in Supplementary Material 1 (see the electronic supplementary material). Additionally, a hand-search using the same search strategy was performed in Google Scholar to identify additional studies.

##### Inclusion and Exclusion Criteria

We considered studies meeting the following criteria: (1) any kind of population, (2) *V*O_2max_ estimated through consumer wearable devices and measured with the reference standard (a graded exercise test to exhaustion with direct or indirect [gas analysis] calorimetry using a mode of test that involves large muscle groups), and (3) criterion validity studies.

We excluded studies following these criteria: (1) non-consumer wearable devices (e.g., research-based accelerometers), (2) not original articles (e.g., reviews or editorials) and grey literature (e.g., meeting abstracts), and (3) articles validating new algorithms in the estimation of *V*O_2max_ that are not yet incorporated in any commercial brand.

#### Study Selection

Two authors (PM-G and HLN) independently performed both the title, abstract, and full-text screening of potential articles and any discrepancy was solved in a consensus meeting with a third author (MS). This systematic review process was performed using the Covidence software (www.covidence.org; Veritas Health Innovation).

#### Data Extraction

For each included article we extracted the following information: (1) author’s name and publication year, (2) target population (e.g., healthy adults), sample size, and age range, (3) protocol used for the *V*O_2max_ assessment via reference standard (e.g., indirect calorimetry), (4) gas analyzer brand used, (5) wearable device used, (6) protocol followed for the estimation of *V*O_2max_ via wearable devices, and (7) statistical analysis used to test the validity of wearable *V*O_2max_ against the reference standard. Two independent authors (PM-G and HLN) performed the data extraction, and any discrepancies were discussed until consensus was reached.

#### Risk of Bias

The Consensus-based Standards for the selection of health Measurement Instruments (COSMIN) checklist was adapted and used to assess the risk of bias of included studies. The COSMIN checklist contains standards for evaluating the methodological quality of studies validating health measurement instruments [22], and it encompasses four domains: (1) participants included, (2) index measure (i.e., wearable device), (3) reference standard (i.e., indirect calorimetry), and (4) statistical analysis. Each domain contains several items with three possible answers (“yes,” “unclear,” and “no”) according to the fulfillment of the criterion and, therefore, the presence or absence of bias (Supplementary Material 2; see the electronic supplementary material). According to the Risk of Bias 2 (RoB 2) criteria proposed by Cochrane [23], an article having at least one “no” or more than two “unclear” items was categorized as having “high risk” of bias; having one “unclear” item was categorized as “some concerns” in the risk of bias; and having all items answered as “yes” was categorized as “low risk” of bias. Two independent researchers (PM-G and AG) accomplished this process, and disagreements were discussed to reach a consensus including a third author (FBO).

#### Meta-Analysis

We identified two main methodologies to estimate *V*O_2max_ through wearable devices: (1) the resting conditions that evaluate users lying in a supine position and/or standing still, and (2) exercise-based methodologies that evaluate users while performing physical activity. Therefore, we performed and reported the meta-analysis separately for these two methods—the resting and exercise tests. The bias of the estimation of *V*O_2max_ by the wearables (i.e., the mean difference between the wearable and the reference standard) and the standard errors of this bias in all included studies were used to calculate the pooled bias and its 95% confidence interval (CI) for both the resting and exercise test. A negative bias represents an underestimation of the wearable *V*O_2max_ relative to the reference *V*O_2max_, while a positive value represents an overestimation. The Higgins *I*^2^ statistic and *P* value were used to test the heterogeneity of included studies, which were classified as not important (0–40%), moderate (30–50%), substantial (50–75%), or considerable (75–100%) [24]. Due to the presence of considerable heterogeneity in both meta-analyses (Higgins *I*^2^ = 77% and 88% in resting and exercise test, respectively), we used a random-effects model of the inverse variance method. Klepin et al. [25] averaged the gas exchange data every 15 and 60 s, and we selected the 15 s time averaging according to previous recommendations [26]. Two studies examined the wearable validity separately in men and women [27, 28], and we maintained this division when including the data in the meta-analysis. There were five studies [29–31] that did not report the bias to test the validity or reported it in plots. Therefore, validity was estimated from correlation coefficients between the wearable and reference *V*O_2max_, as suggested elsewhere [32], or extracted from plots through the WebplotDigitizer software (Ankit Rohatgi, website: https://automeris.io/WebPlotDigitizer/), which has demonstrated an excellent validity and reliability in extracting graphed data [33].

The framework for the meta-analysis of Bland–Altman studies proposed by Tipton and Shuster [34] was used to obtain a pooled limit of agreement in both the resting and exercise test, which was calculated with the following formula: *δ* ± 2 √ *σ*^2^ + *τ*^2^, where *δ* is the average bias across studies, *σ*^2^ is the average within-study variation in differences, and *τ*^2^ is the variation in bias across studies [34]. The weighted least-squares models from the abovementioned random-effect meta-analysis were used to estimate *δ* and *σ*^2^, while the DerSimonian and Laird procedure was used to estimate *τ*^2^ [35]. The R code provided in the study of Tipton and Shuster [34] was used to conduct all these analyses with the RStudio statistical program.

Three sensitivity analyses were performed: (1) to test the robustness of the results, (2) to evaluate the presence of publication bias, and (3) to divide the meta-analyses results into those studies using photoplethysmography (PPG) technology to assess HR versus those using chest straps. For the robustness analysis, studies were removed one at a time and we tested whether the overall effect size (i.e., *z* score and *P* value) was significantly modified in magnitude or direction. The publication bias was assessed by a funnel plot and the Egger regression asymmetry test, considering the level of significance < 0.100 [36]. The meta-analysis was repeated in the two following conditions: (1) splitting the results into studies using PPG and chest straps to measure HR and (2) including studies from the last 3 years. Thus, we tested the impact of the different types of HR recordings (PPG vs. chest straps) and of old articles testing obsolete devices on the error estimates.

The meta-analysis was performed using the Review Manager Version 5.3 (The Nordic Cochrane Center, The Cochrane Collaboration, 2014, Copenhagen, Denmark), and the limit of agreement meta-analyses were performed using the RStudio statistical program (version 1.4.1106, R Core Team 2020; R Foundation for Statistical Computing, Vienna, Austria; https://www.R-project.org/).

## Results

### Summary of the Included Studies in the Systematic Review

**Fig. 1:**
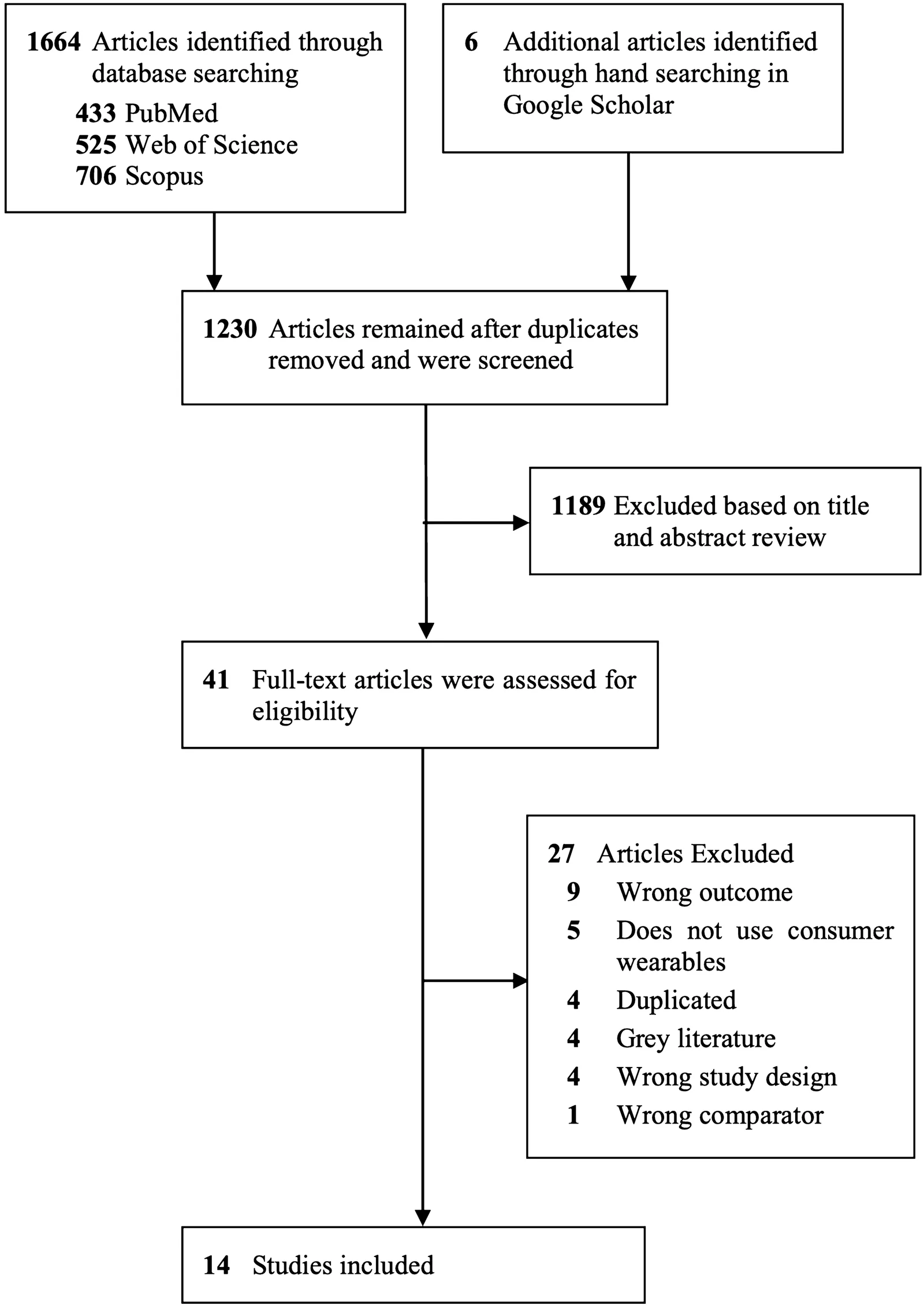
Flowchart of the systematic review process

**Fig. 2:**
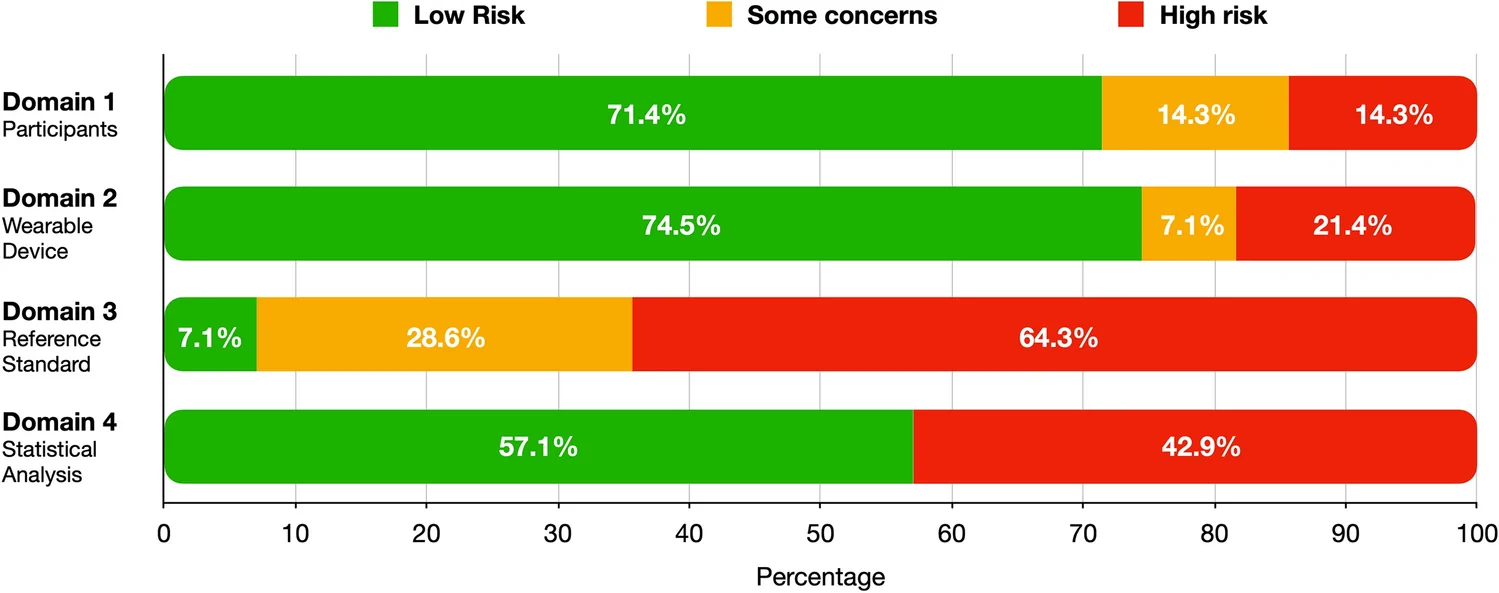
Risk of bias assessment divided by domains

The flow chart (Fig. 1) shows that among the 1224 non-duplicated studies initially included, 1189 were excluded after the first screening of title and abstract and another 27 were further excluded after the full-text screening. Consequently, 14 articles meeting the inclusion criteria were included in the systematic review and the meta-analysis; eight and eight studies reporting on the validity of an exercise-based and resting state-based methodology, respectively, were included. Table 1 summarizes the main information extracted from the 14 included studies, including a total of 403 participants. The risk of bias assessment of included studies is reported in Fig. 2 and Supplementary Material 3 (see the electronic supplementary material). The overall risk of bias assessed across all domains was deemed to be “some concerns” for three (21%) and “high” for 11 (79%) of the 14 studies included.

**Table 1:** Characteristics of included studies (*N* = 14)

References	Participants	Age (years)	Wearable device. HR assessment	Setup information	*V*O_2max_ estimation	Reference standard	*V*O_2max_ protocol	Statistical analysis
Anderson et al. 2019 [29]	25 recreational runners, men (17) and women (8)	39.4 ± 10.8	Garmin Fenix 5X. Wrist-measured HR (PPG)	Age, sex, height, and weight	Exercise test: walking or jogging warm-up + 10-min run at their highest perceived pace + 5-min cool down walking	Indirect calorimetry: ParvoMedics TrueOne 2400	Treadmill: Bruce running protocol (speed and inclination increase each 3 min)	*T* test and Pearson’s *r*
Carrier et al. 2020 [44]	17 recreational runners, men (8) and women (9)	24.8 ± 4.3	Garmin Fenix 3 + chest HR strap	HR_max_ and unspecified info	Exercise test: 15-min outdoor run above 70% HR_max_	Indirect calorimetry: ParvoMedics	Treadmill: modified Costill-Fox running protocol (speed increase first and 2% inclination increase second each 2 min)	*T* test, MAPE, Pearson correlation and Bland–Altman
Cooper and Shafer 2019 [47]	19 healthy, men (9) and women (10)	21.9 ± 4.2	Polar A300 + chest HR strap	Age, sex, height, and weight	Resting HR: 5 min supine position	Indirect calorimetry: Cosmed Fitmate Pro	Treadmill: Bruce running protocol (speed and inclination increase each 3 min)	Pearson’s *r* and ANOVA
Crouter et al. 2004 [27]	20 active men (10) and women (10)	Men: 26.0 ± 3.1 Women: 23.0 ± 2.4	Polar S410 + chest HR strap	Age, sex, height, weight, and physical activity level	Resting HR: supine position	Indirect calorimetry: ParvoMedics TrueMax 2400	Treadmill: individual ramp running protocol (individual start, increase 1% incline per min)	*T* test and Pearson’s *r*
Esco et al. 2011 [37]	50 active men	24.0 ± 5.1	Polar F11 + chest HR strap	Age, sex, height, weight, and physical activity level	Resting HR: supine position	Indirect calorimetry: ParvoMedics TrueOne 2400	Treadmill: Bruce running protocol (speed and inclination increase each 3 min)	*T* test, Pearson’s *r* and Bland–Altman
Esco et al. 2014 [40]	20 female soccer players	21.5 ± 1.7	Polar FT40 + chest HR strap	Age, sex, height, weight, and physical activity level	Resting HR: 5 min supine position	Indirect calorimetry: ParvoMedics TrueOne 2400	Treadmill: Bruce running protocol (speed and inclination increase each 3 min)	Bland–Altman and MAPE
Freeberg et al. 2019 [46]	30 healthy, men (17) and women (13)	21.7 ± 3.1	Fitbit Charge 2. Wrist-measured HR (PPG)	Not specified	Exercise test: 2 × 10 min at highest intensity possible	Indirect calorimetry: ParvoMedics TrueOne 2400	Treadmill: individual ramp running protocol (4–7 mph, increase 1% incline per min) + verification test	ANOVA, Pearson’s *r*, MAPE, Bland–Altman and ICC
Klepin et al. 2019 [25]	65 healthy men (27) and women (33)	31.0 ± 7.3	Fitbit Charge 2. Wrist-measured HR (PPG)	Age, sex, handedness, height, and weight	Exercise test: 3 × 15 min at comfortable pace	Indirect calorimetry: Cosmed	Treadmill: ramp running protocol (5 mph, increase by 0.75 MET per min)	Bland–Altman and MAPE
Kraft and Dow 2017 [30]	16 healthy, men (10) and women (6)	22.4 ± 5.2	Garmin Forerunner 920XT + chest HR strap	Height and weight	Exercise test: 10 min self-paced run	Indirect calorimetry: ParvoMedics TrueOne 2400	Treadmill: Bruce running protocol (speed and inclination increase each 3 min)	*T* test
Kraft and Dow 2018 [31]	18 healthy, men (12) and women (6)	21.3 ± 2.2	Polar RS300X + chest HR strap	Age, height, weight, sex, and activity level	Resting HR: 5 min supine position	Indirect calorimetry: ParvoMedics TrueOne 2400	Treadmill: Bruce running protocol (speed and inclination increase each 3 min)	*T* test and Pearson’s *r*
Lowe et al. 2010 [51]	32 active women	20.3 ± 1.9	Polar F6 + chest HR strap	Age, sex, height, and weight	Resting HR: 5 min sitting position	Indirect calorimetry: ParvoMedics	Treadmill: Bruce running protocol (speed and inclination increase each 3 min)	*T* test
Passler et al. 2019 [39]	24 healthy, men (13) and women (11)	23.4 ± 2.1	Polar V800. Wrist-measured HR (PPG)	Not specified	Resting test: 10 min supine position (pretest), 3 min supine position, 3 min standing position	Indirect calorimetry: Metalyzer 3B-R3, Cortex	Treadmill: ramp protocol (7 km·h^−1^, increase by 0.5 km·h^−1^ per min)	*T* test, MAPE, Bland–Altman and ICC
			Garmin Forerunner 920 XT. Wrist-measured HR (PPG)	Not specified	Exercise test: > 10 min self-paced run			
Snyder et al. 2019 [28]	44 healthy, men (22) and women (22)	Men: 24.7 ± 5.4 Women: 25.0 ± 4.3	Polar V800 + chest HR strap	Age, sex, height, weight, and physical activity level	Resting HR: 5 min supine position	Indirect calorimetry: ParvoMedics TrueOne 2400	Treadmill: Bruce running protocol (speed and inclination increase each 3 min)	ANOVA, Bland–Altman and Pearson’s *r*
			Garmin Forerunner 230 + chest HR strap	Age, sex, height, weight, and HR_max_	Exercise test: 10 min self- paced run			
Wagner et al. 2020 [42]	23 healthy men	23.1 ± 2.5	Garmin GF5		Exercise test: 10 min and 30 s all out run	Indirect calorimetry: Metalyzer 3B, Cortex	Treadmill: ramp running protocol (10 km·h^−1^, incline 5%, increase by 2.5% per min)	Bland–Altman and ICC

*ANOVA* analysis of variance, *HR* heart rate, *HR*_*max*_ maximum heart rate, *ICC* intraclass correlation coefficient, *MAPE* mean absolute percentage error, *MET* metabolic equivalent, *PPG* photoplethysmography, *VO*_*2max*_ maximal oxygen consumption

### Validity of the *V*O_2max_ Estimated by Wearables: Meta-Analysis

**Fig. 3:**
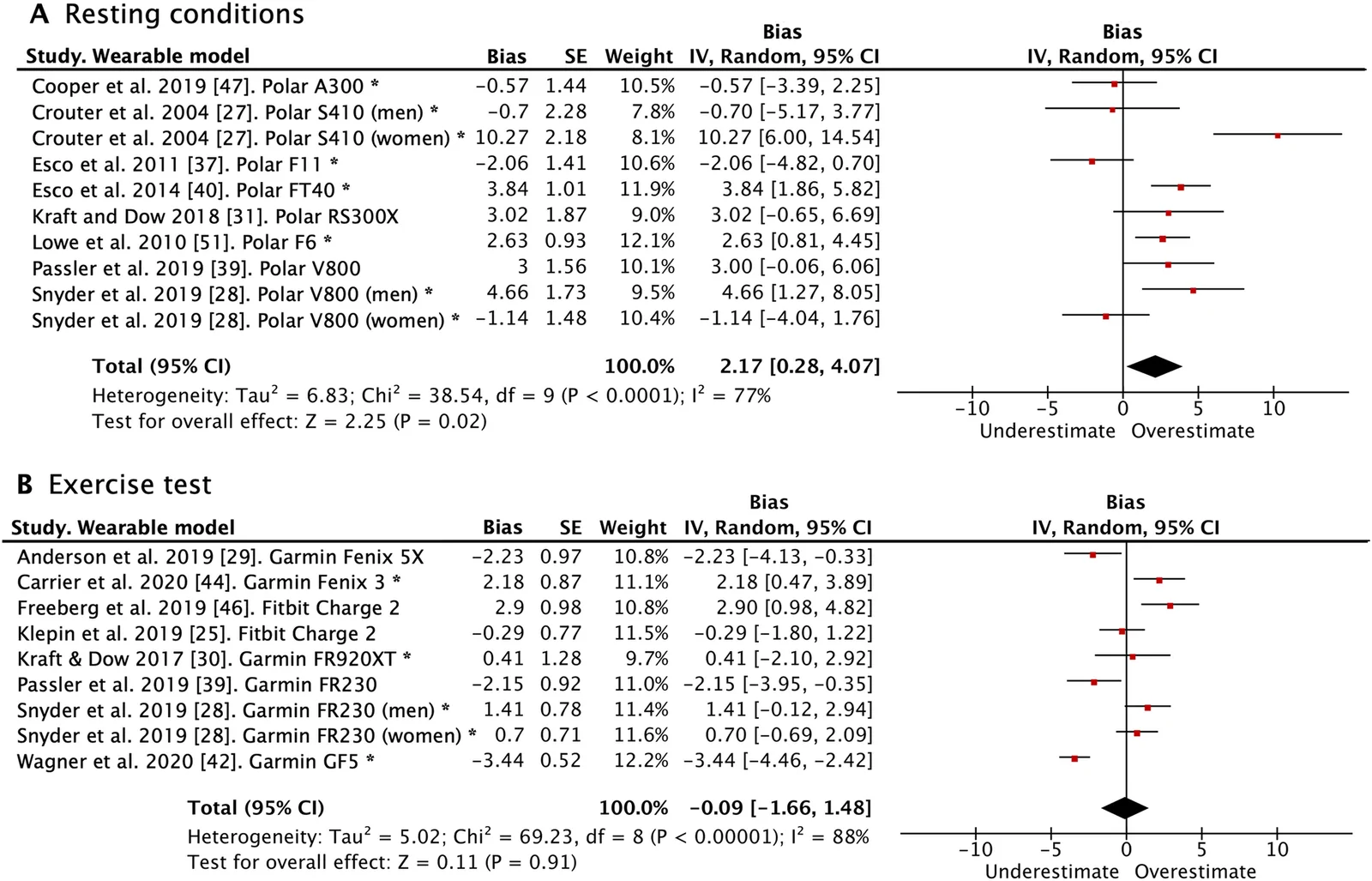
Pooled bias and SE for wearables *V*O_2max_ using resting conditions (**A**) and exercise tests (**B**) relative to the reference standard. A negative bias represents an underestimation and a positive bias an overestimation of the *V*O_2max_ estimated from wearables in comparison to the reference standard. *CI* confidence interval, *SE* standard error, *VO*_*2max*_ maximal oxygen consumption. *Heart rate was measured with chest strap. In the remaining articles not flagged with an asterisk, heart rate was measured using photoplethysmography technology on the wrist

The forest plots with the pooled bias between the reference *V*O_2max_ and the wearable estimation are presented in Fig. 3 for both the wearables using the resting methodology and the exercise test. Wearables using the resting test significantly overestimated *V*O_2max_ (bias = 2.17 ml·kg^−1^·min^−1^; 95% CI 0.28–4.07; *P* = 0.020) in comparison to the reference standard. On the other hand, wearables estimating *V*O_2max_ through exercise tests showed a bias close to nil compared to the reference standard (bias =  − 0.09 ml·kg^−1^·min^−1^; 95% CI − 1.66 to 1.48; *P* = 0.910). Sensitivity analysis showed a lack of robustness in the resting test meta-analysis since results were significantly modified when removing five individual studies [27, 28, 37–39], while the exercise test meta-analysis indeed demonstrated robustness (Supplementary Material 4; see the electronic supplementary material). After a visual observation of the funnel plot and confirming with the Egger’s tests, we did not find evidence of publication bias either in the resting test or exercise test studies (Supplementary Material 5). Studies using PPG technology in the HR recording had significantly greater bias than those using chest strap in resting conditions, while the difference was not statistically significant in the exercise testing methodology (Supplementary Material 6 and 7). Finally, we excluded five articles from more than 3 years ago in the resting conditions and we observed a significant reduction in the estimation errors (bias = 1.66 ml·kg^−1^·min^−1^; 95% CI − 0.58 to 3.90; *P* = 0.150).

**Fig. 4:**
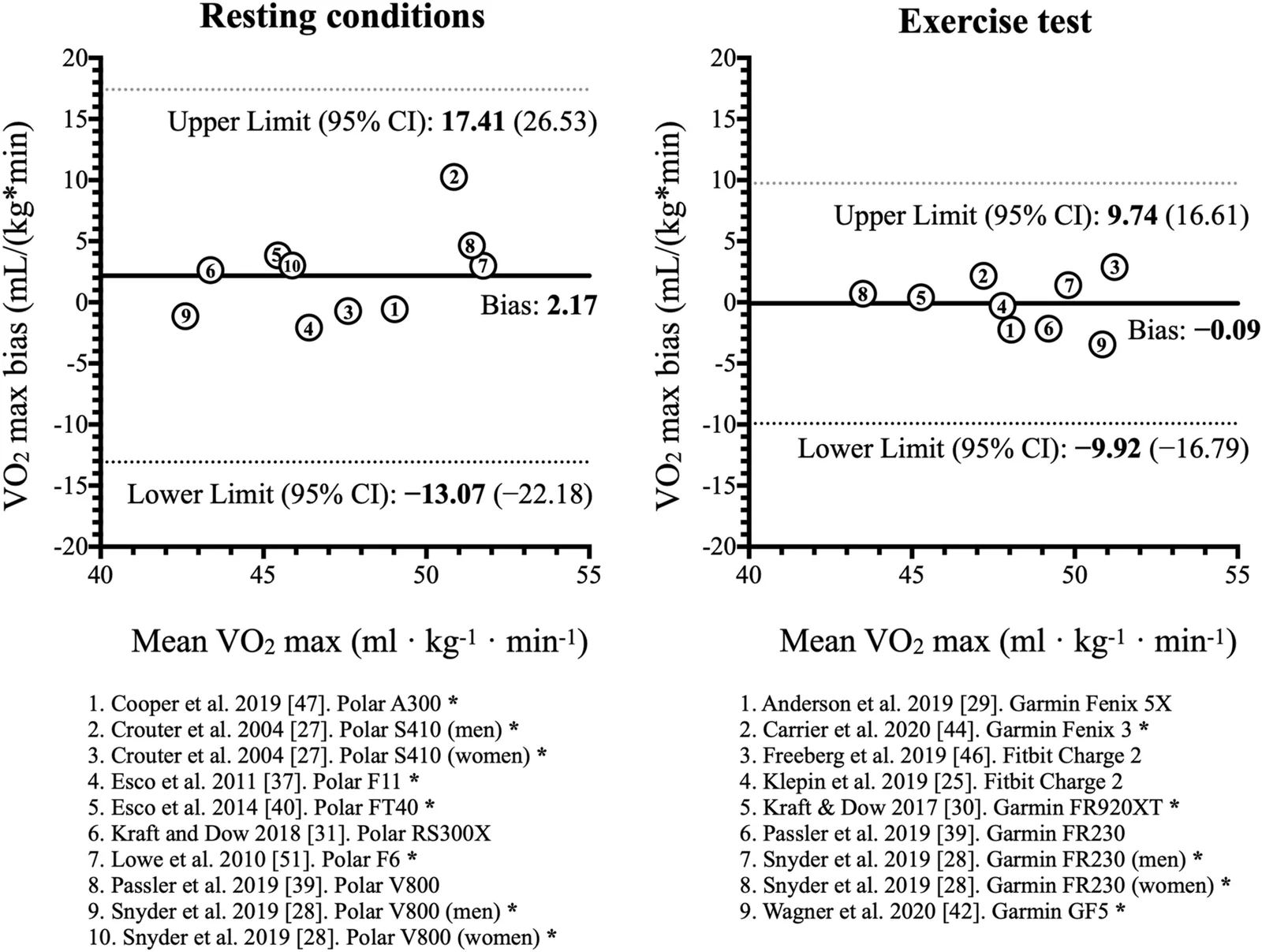
Bland–Altman meta-analysis for the comparison of wearable-derived *V*O_2max_ using resting conditions and exercise tests with the reference *V*O_2max_. The *y*-axis is the bias between the wearable and reference *V*O_2max_ (wearable − reference), with positive values indicating an overestimation and negative values an underestimation by the wearable. The *x-*axis is the mean *V*O_2max_ between the wearable and reference. *CI* confidence interval, *VO*_*2max*_ maximal oxygen consumption. *Heart rate was measured with chest strap. In the remaining articles not flagged with an asterisk, heart rate was measured using photoplethysmography technology on the wrist

The Bland–Altman plot (Fig. 4) presents the pooled bias and its limits of agreement for both the resting and exercise methodologies. The limits of agreements in the resting test spanned from − 13.07 to 17.41 ml·kg^−1^·min^−1^ (i.e., ± │15.24│; 95% CI − 22.18 to 26.53), while limits were narrower in the exercise tests, spanning from − 9.92 to 9.74 ml·kg^−1^·min^−1^ (i.e., ± │9.83│; 95% CI − 16.79 to 16.61). Therefore, the difference in limits of agreement was smaller by 5.4 ml·kg^−1^·min^−1^ in exercise tests compared to the resting conditions. The limits of agreement in the different studies using the resting conditions ranged from ± 17.75 [40] to ± 38.97 ml·kg^−1^·min^−1^ [41], while it spanned from ± 11.18 [42] to ± 23.53 ml·kg^−1^·min^−1^ [25] in the exercise tests. Lastly, studies using PPG technology in the HR recording had a greater span of the limits of agreement in comparison with those using chest strap in the exercise tests (± 23.03 vs. ± 17.97 ml·kg^−1^·min^−1^). It was not possible to make a comparison in the resting conditions due to only two studies using PPG.

### The Current State of Knowledge in Validation Protocols Relevant to Inform Best-Practice Recommendations

**Fig. 5:**
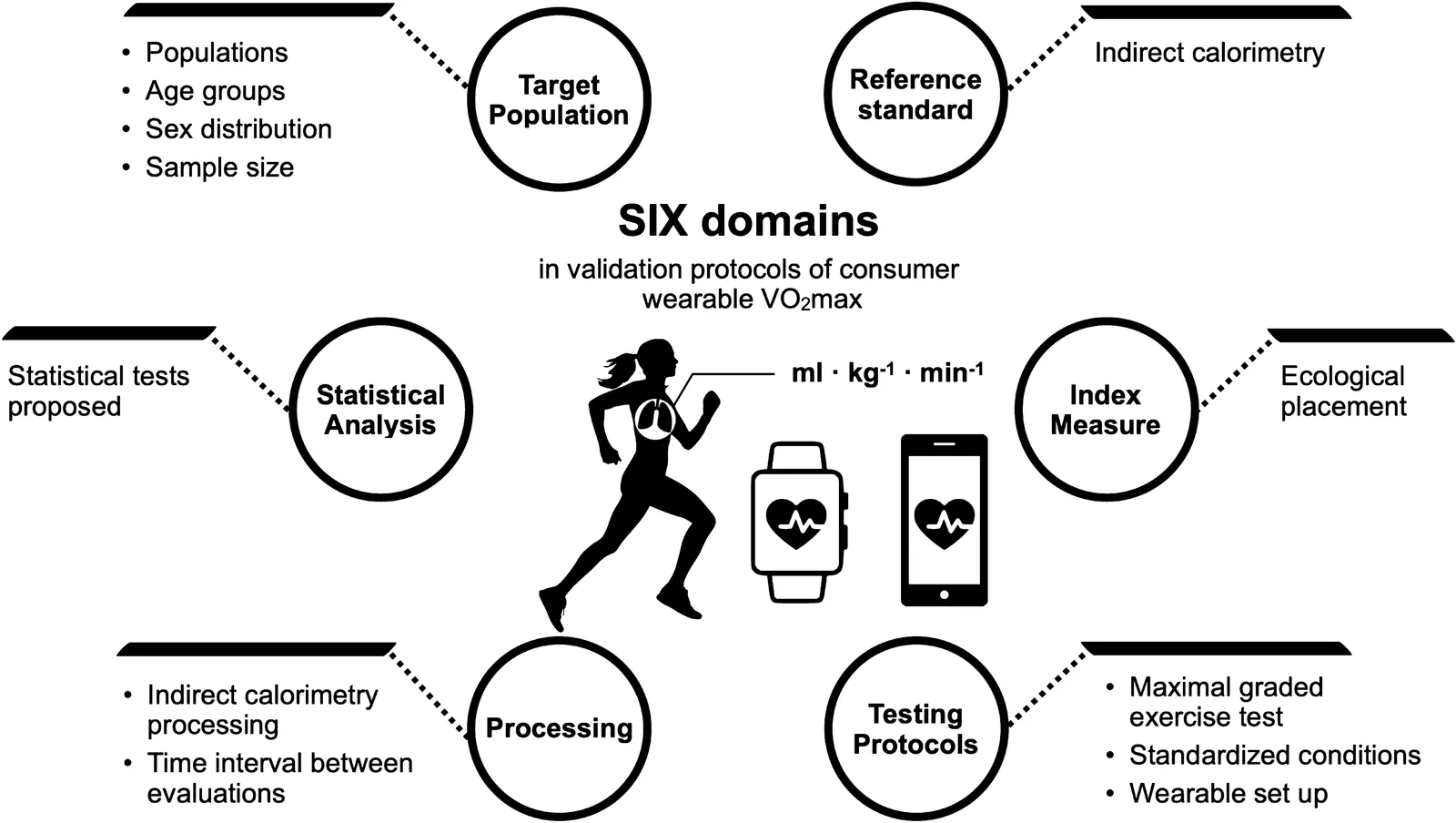
Six domains and corresponding variables of interest identified as being of importance in the validation of consumer wearable estimation of *V*O_2max_. *VO*_*2max*_ maximal oxygen consumption

Similar to the previous statements of the INTERLIVE consortium [19, 20], we present and discuss the information found in these studies divided into the six key domains to take into consideration when designing validation protocols of consumer wearables estimating *V*O_2max_ (Fig. 5).

#### Target Population

The total sample size studied was 403 participants (218 men and 185 women), with a mean sample per article of 29 participants. For future validation studies, we recommend performing a priori sample size calculation following the approach by Lu et al. [43], which uses the Bland–Altman limit of agreement analysis. The required sample size to obtain a power of 80–90% is calculated considering the expected mean absolute difference between the index measure and the reference standard, the expected SD of this difference, and the maximum allowed difference predefined by the researchers. It is advised to conduct a pilot study to obtain this information directly from the devices to be validated. If this is not feasible, our meta-analysis reveals that the expected mean absolute difference in the resting conditions is 2.30 ml·kg^−1^·min^−1^ and the expected SD is 7.20 ml·kg^−1^·min^−1^, whereas the expected mean absolute difference in the exercise test is 1.32 ml·kg^−1^·min^−1^ and the expected SD is 4.03 ml·kg^−1^·min^−1^. Regarding the maximum allowed difference, there is no agreement on this size with respect to relevance for performance, health promotion, or clinical practice. In the second paragraph of the “Discussion” section, we argue the potential meaningfulness of the estimation errors by wearables considering previous meta-analyses on *V*O_2max_ changes and mortality risk. However, it is important to know that this maximum allowed difference must be greater than the expected mean difference ± 1.96 × the expected SD. Thus, considering our meta-analysis results, these values should be at least 16.41 and 9.22 ml·kg^−1^·min^−1^ in the resting conditions and exercise test, respectively. Raising the sample size will not affect the estimated size of the limit of agreement but will provide greater precision (i.e., tighter confidence bands around the limit of agreement).

Participants from the included studies were adults with a pooled age of 24.6 ± 5.7 years old. However, children, adolescents and older adults also use these wearable devices in real life, and, therefore, we recommend that future validation studies include different age populations to ensure that the validity is representative of the general population. Regarding sex differences, Crouter et al. [27] found a remarkably larger error when estimating *V*O_2max_ in women compared to men, while Snyder et al. [28] showed opposite results, with a greater error in men compared to women. We suggest future studies to test whether the validity of existing methods/algorithms systematically differs according to sex.

In the risk of bias assessment, we identified that the majority of articles (10 of 14) adequately delimited the target population they wanted to study and nearly all participants contributed with data to be included in the validity analysis. Participants from the included studies were all physically active people categorized as “healthy” or “active,” recreational runners [29, 44] or soccer players [40]. In order to have a wider representation of the general population, *V*O_2max_ estimations from consumer wearables should be tested in further clinical populations such as old adults, individuals with more sedentary behaviors, with overweight/obesity, or highly trained athletes. We, therefore, recommend expanding the population included beyond healthy young people (e.g., from very untrained sedentary people to highly trained athletes), as well as to clearly define and report the inclusion/exclusion criteria used to define these target populations.

### Reference Standard

All studies included indirect calorimetry through gas analysis as a reference standard of *V*O_2max_, as was previously recommended [45]. In brief, indirect calorimetry measures *V*O_2_ and VCO_2_ concentrations and calculates the respiratory exchange ratio (RER), allowing for the obtainment of *V*O_2max_ while exercising [45]. The gas analysis systems used were reported in all studies, where Parvo Medics was the most popular brand, used in ten studies [27–31, 37, 38, 40, 44, 46], followed by Cosmed [25, 47] and Metalyzer [39, 42], with two studies each. Although the validity and reliability of indirect calorimetry systems may seem obvious, available devices are not always reliable [48, 49] and only one of the included studies provided a reference with regards to the validity within the study [29]. Similarly, only two studies included in this review specified whether the gas exchange was recorded breath by breath [39, 42]. Furthermore, none of the included articles reported whether the gas analyzer used both *V*O_2_ and *V*CO_2_ for *V*O_2max_ assessment, even though it is known that systems without CO_2_ sensors decrease the precision and should be treated with caution [50]. Lastly, four studies [39, 42, 44, 47] did not clarify whether the device was calibrated [45], and we recommend that a proper calibration process according to the manufacturer’s instructions be performed before the *V*O_2max_ assessment. We urge authors and developers to improve transparent reporting by including at a minimum the brand used, the type of recording technology (e.g., breath by breath or mixing chamber), and previous validity/reliability of the instruments.

### Index Measure

Within the included studies in this review, eight validated the *V*O_2max_ estimations of Polar^®^ devices (models: A300, S410, F11, FT40, F6, RS300X, and two V800) [27, 28, 31, 37, 39, 40, 47, 51], five validated Garmin^®^ devices (models: Fenix 3, Fenix 5X, Forerunner 920 XT, and GF5) [29, 30, 39, 42, 44], and two validated Fitbit^®^ devices (models: two Charge 2) [25, 46]. However, several other brands currently claim to provide *V*O_2max_ estimations, such as Apple, TomTom, Huawei, Suunto, Withings, and Coros (Supplementary Material 8; see the electronic supplementary material). Considering that scientific validation of these devices is lacking, we suggest future validity studies on these remaining brands in order to improve transparency.

Three out of the 14 included studies did not follow an ecological validity procedure [28, 29, 44], defined as a validation process that resembles the use of the device in the consumer’s real life. Two of the studies introduced bias when including the setup information, an aspect that will be discussed in the “Testing Protocols and Conditions” section [28, 44], while one study did not place the device in an ecological manner according to manufacture instructions [29]. Regarding the ecological placement, Anderson et al. [29] fixed the device to the wrist with additional tape, and this is not recommended since it may artificially improve the precision of the HR readings through PPG, biasing the validity of the device in ecological settings. Overall, we recommend that wearable devices be worn on ecological body locations in accordance with the manufacturer’s instructions, and this location should be adequately described within the methods. If multiple wrist-worn devices are being tested, a maximum of two devices per wrist should be used at the same time, with placement being randomly counterbalanced between participants.

Apart from the wrist-worn wearables, nine devices incorporated a chest strap to record HR during the *V*O_2max_ estimation [28, 30, 37, 38, 40, 44, 47]. Chest-strap technology has been the most used method for HR monitoring in the past. Moreover, it is widely accepted as a valid and reliable method to measure HR in free-living conditions, but it presents limitations in 24 h recording over multiple days. Recently, many wearables are built with the possibility to measure HR at the wrist using the PPG technology, which allows longer recording time and a more comfortable measurement by not incorporating additional devices along with the wrist bracelet (e.g., chest strap). A recent meta-analysis has also revealed an acceptable validity of the PPG technology during treadmill running and walking (mean difference − 0.51 bpm; 95% CI − 1.60 to 0.58 bpm), yet an underestimation when performing endurance sports (mean difference − 7.26 bpm; 95% CI − 10.46 to − 4.07 bpm) [52]. Therefore, the type of HR measurement is relevant and should be reported in the validation protocols. Future research is necessary to determine whether the *V*O_2max_ estimation is more accurate using the HR obtained by PPG or chest strap. Furthermore, the validity of HR measures from wearables should be tested before being used in the *V*O_2max_ estimation following the recently published recommendations by the INTERLIVE consortium [19].

### Testing Protocols and Conditions

#### Reference Standard

All of the included studies tested *V*O_2max_ in laboratory conditions. The two previous expert statements of the INTERLIVE consortium on step count and HR provided recommendations for semi-free-living and free-living conditions besides the laboratory setting to test the ecological validity [19, 20]. However, reference *V*O_2max_ is still recommended to be performed in laboratory conditions, and, therefore, the free-living and semi-free-living conditions do not apply in this context. Regarding the type of activity, all included studies applied treadmill running protocols. It is known that running protocols may provide small differences in *V*O_2max_ in comparison to cycle protocols [53], and, therefore, our recommendation is to incorporate protocols that are as close as possible to the type of activity for which the consumer wearable has been designed.

In regards with the work rate progression, some protocols gradually increased the speed [25, 39], the treadmill inclination [27, 42, 46], or both intensity conditions within the protocol [28–31, 40, 41, 44, 47, 51]. Five studies used ramp protocols [25, 27, 39, 42, 46] in which work rate increases more gradually (e.g., each 30–60 s), while the remainder studies included blocks of 2 [44] or 3 min [28–31, 37, 40, 47, 51]. It seems that *V*O_2max_ does not vary whether treadmill inclination or speed increase is used [53]. Likewise, the use of a ramp versus a more accentuated increase in the work rate does not affect the *V*O_2max_ measure, although each progression has pros and cons depending on the target population and whether treadmill or cycle ergometer is used [54]. We recommend selecting an appropriate work rate progression according to the type of population in which the consumer wearable is intended to be validated and the selected physical activity (e.g., running or cycling).

Maximal graded exercise testing requires participants to terminate the test at volitional fatigue, and accepted criteria exist to ensure that maximal *V*O_2_ during the test was reached. For more information, we refer readers to chapter 4 of the American College of Sports Medicine’s (ACSM’s) Guidelines for Exercise Testing and Prescription, in which a detailed description of test termination criteria can be found [7]. Among the included studies, five did not consider at least two maximum-effort criteria apart from voluntary exhaustion and are likely to have measured *V*O_2peak_ instead of *V*O_2max_ [25, 30, 31, 39, 44]. In the last years, an alternative/complementary solution named “verification phase” has been proposed, which includes an extra effort lasting between 2 and 3 min at a supramaximal work rate (i.e., 110% of maximum power) after the test termination to corroborate the results [55]. This approach was only followed by Freeberg et al. [46] and may be an interesting method to use in future validation protocols.

A maximal graded exercise test normally requires several standardized conditions to ensure that the participants reach their true *V*O_2max_. Five out of the 14 included articles considered at least some of these standardized conditions before the exercise testing [27, 29, 38–40], whereas the remainder did not report this information. The INTERLIVE consortium recommends taking into account the following standardized conditions when measuring the *V*O_2max_ reference standard: caloric uptake, caffeine or alcohol consumption, intensive sports activities, medications, and an appropriate warm-up (e.g., 5–10 min of light-intensity aerobic exercise and dynamic stretching) before commencing the exercise test [7, 53].

#### Wearable Device

Included studies that estimated *V*O_2max_ from a resting test were Polar devices and the test used was the patented “Polar fitness test” [56]. Polar devices record the resting HR and heart rate variability (HRV) via Polar chest strap or the PPG technology incorporated into the device and use these data to estimate *V*O_2max_ [57]. This protocol slightly differed based on the wearable model, but always ranged from 5 to 10 min in a supine position (e.g., Polar A300, FT40, and F6), while only one of the included models additionally added a few minutes in a standing position (e.g., Polar V800). On the other hand, only Garmin and Fitbit were the brands that used exercise testing. The Fitbit exercise test consists of a run at a comfortable pace for at least 10 min while the GPS is being recorded [58]. Garmin devices offer different methods to estimate *V*O_2max_ depending on three types of activity: running, cycling, or walking [59]. However, only the running protocol was used in all studies included in this review [28–30, 42, 44], requiring a run of at least 10 min, while recording the GPS signal and HR data (through PPG technology or chest strap). Garmin’s instructions recommend an intensity of at least 70% of the user’s maximal HR for the entire exercise, which can be either estimated or manually input by the user [59]. Overall, we recommend researchers systematically follow the manufacturer’s recommendations when estimating *V*O_2max_ from the wearable device among study participants.

Some of the included wearable devices require a previous setup in which personal data such as age, sex, height, weight, or physical activity level are recorded to improve the accuracy of the *V*O_2max_ estimation. Only two of the included studies did not specify whether previous setup information was input prior to commencing the validation protocol [39, 46], while the remainder of the studies recorded some basic information. As a general recommendation, all the setup information required by the device should be included and reported, and this should be similar to the information customers are provided outside of a research context. For instance, both Snyder et al. [28] and Carrier et al. [44] introduced the maximum heart rate (HR_max_) obtained from the reference standard test into the consumer wearables, which is not ecological since few users have HR_max_ data from a maximal graded exercise test in laboratory conditions.

### Data Processing

#### Reference Standard

Indirect calorimetry for either mixing-chamber or breath-by-breath technology requires several decisions on data processing while conducting *V*O_2max_ tests. A major factor for removing variability in indirect calorimetry is the time and breath averages used to estimate *V*O_2max_. Only three [25, 27, 46] of the studies included in this review reported this relevant information. Following Robergs et al. [26] recommendations, between 15 and 30 s time averages and 15-breath running averages should be used to have a reasonable reduction in data variability without losing relevant physiological information. For researchers implementing digital filters, a low cut-off frequency of 0.04 Hz is recommended [26].

#### The Time Interval Between Evaluations

With regards to wearable devices, modifying data processing is not possible since the wearables directly compute the *V*O_2max_ using algorithms that are usually proprietary information and the exact equations are not disclosed. An important consideration, however, is the time interval between both assessments, since the fatigue after the maximal exercise test may affect the wearable *V*O_2max_ estimation. Since the resting methodology is conducted in resting conditions, these wearable protocols can be performed before the reference standard protocol without influencing either test. This should not be performed in the opposite order, since the maximal test required for the reference standard could affect the resting HR or HRV. Concerning the wearable estimations based on the exercise test, 24–48 h between tests is recommended to ensure optimal recovery from high-intensity exercise and avoid associated muscle fatigue hampering the performance [60]. Furthermore, randomization or counterbalancing the order of the wearable and laboratory tests is important to control the potential carryover effects. Five of the included studies in this review either did not meet this time-interval criterion or did not report any information [25, 28, 29, 39, 42], and none mentioned any randomization or counterbalancing strategy, which is an aspect to consider in future validation studies.

### Statistical Analysis

The Bland–Altman limits of agreement analysis is the most popular method used in validation studies and has been widely accepted as the most appropriate type of statistical analysis in these types of studies [61, 62]. In brief, Bland–Altman analysis provides both the systematic error (i.e., bias or average difference between methods) and the random error or precision (i.e., 95% limit of agreement of the systematic error), thus providing valuable information for the comparison of the wearable devices to the reference standard. The lower and upper bound of the limits of agreement provides an estimate in which 95% of future observations of the differences in *V*O_2max_ between the wearable device and a criterion reference assessment are expected to fall. In addition, the Bland–Altman plots represent the individual difference between methods against the mean of the methods, providing visual information on other relevant dimensions of agreement, such as heteroscedasticity (a trend to increase/decrease the error between methods as the magnitude of the measurement increases). Additionally, percentage error measures, such as the mean absolute percentage error (MAPE), represent a helpful option to report the error of the device in an easy-to-understand manner [63]. Therefore, we recommend reporting percentage error measures complementary to the limit of agreement analysis. In the risk of bias assessment, we detected that five studies did not apply an appropriate analysis of agreement between the wearable devices and the reference standard, since they only performed mean difference (*t* test or analysis of variance [ANOVA], but did not report the limits of agreement or the Bland–Altman plots) or Pearson correlation analyses [27, 29–31, 47, 51]. Among the statistical tests used, Bland–Altman [25, 28, 37, 39, 40, 42, 44, 46], *t* test [27, 29–31, 37–39, 44], and Pearson’s *r* [27–29, 31, 37, 44, 46, 47] were the most popular tests, with eight studies using each of these analyses, followed by MAPE in five studies [25, 39, 40, 44, 46] and intraclass correlation coefficient [39, 42, 46] or ANOVA [28, 46, 47] in three studies each.

The last point to consider is the contextual validity of wearable devices in estimating *V*O_2max_, which should be considered within the statistical analysis. For instance, if a wearable device is designed to monitor *V*O_2max_ changes that improve users’ health, the systematic and random errors should be critically analyzed to ensure that the device is capable of detecting individual changes, which are considered clinically significant in the scientific literature. We have already proposed in the “Methods” section that 3.5 and 1.75 ml·kg^−1^·min^−1^ might be potential thresholds since both are normal *V*O_2max_ changes in the general population and have been associated with health improvements. Therefore, companies should report the level of error in a transparent manner according to the purpose of the device and the target population. This would guide researchers in the statistical analysis and the interpretation of the results.

### Recommended Validation Protocol

Based on the abovementioned state of knowledge and the critical discussion between the members of the INTERLIVE consortium, we present best-practice recommendations for validation protocols of *V*O_2max_ derived from consumer wearable devices in Table 2. Furthermore, a checklist is provided in Table 3, including the items to be considered when planning validation protocols of *V*O_2max_ consumer wearables. A graphical overview of the six domains to consider in these validation protocols is presented in Fig. 5.

**Table 2:** The proposed best-practice protocols for the validation of wearable-derived *V*O_2max_

Domain	Variable	Protocol consideration	Reporting consideration
Target population	Population	If purpose is to validate wearable-derived *V*O_2max_ for the general healthy population, a broad heterogeneous sample should be used If purpose is to use wearables in specific clinical applications, validation should be performed in homogenous samples	Report the inclusion/exclusion criteria defining the target population and recruitment methodology and provide basic demographic information (e.g., age, height, weight, or BMI)
	Age	Validation protocols targeting a general healthy population should include the main age ranges: children (< 12 years), adolescents and adults (13–64 years), and older adults	Average and range of sample age should be reported
	Sex	Include an equal sample of males and females within the study	The number of female and male participants should be reported
	Sample size	For those studies aimed at testing the accuracy of a given device, a sample size calculation should be performed based on the previously published data according to Lu et al.[43]. If no previous data are available or this is not the focus of the evaluation, we advise to include a minimum of 15 participants per age group according to previously published recommendations on wearables-derived health measures [19, 20]	Describe the sample size calculation if included If sample size calculation is not feasible, cite previous literature supporting the inclusion of a recommended sample size Describe the flow of sample size recruited and analyzed
Reference standard	Indirect calorimetry	The gold standard for the assessment of *V*O_2max_ is a maximal graded exercise test, performed in laboratory conditions with indirect calorimetry [7] Any brand of metabolic cart is accepted when reporting validity and reliability, as well as measuring both *V*O_2_ and *V*CO_2_ during expiration The metabolic cart should be properly calibrated before the *V*O_2max_ assessment according to manufacturer’s instructions	Indicate if indirect calorimetry was used Report the metabolic cart used, the type of recording technology (e.g., breath-by-breath), and whether the metabolic cart used is valid and reliable Describe the calibration process of the metabolic cart
Index measure	Wearable devices	Consumer wearables should be worn in ecological body locations in accordance with the manufacturer’s instructions. If wrist worn, a maximum of 2 devices per wrist should be used at the same time, with placement being randomly counterbalanced between participants Wearable devices can measure HR with PPG and/or chest-strap technology, and this may have an impact on the *V*O_2max_ estimation	Report the placement of the device and information on order of placement if more than one wrist worn device is used Specify whether HR was recorded with PPG on wrist/arm (or others) or chest-strap technology
Testing protocols and conditions for both reference and index measure	Maximal graded exercise testing with indirect calorimetry	The accepted protocol to assess *V*O_2max_ is a maximal graded exercise testing evaluated in laboratory conditions Maximal test requires participants to perform to the point of volitional fatigue, and at least two accepted criteria are recommended to ensure that participants are reaching the maximum effort during the tests. The ACSM proposes several maximum-effort criteria that can be used [7] A verification phase after the maximal test is recommended to compare both *V*O_2max_ results. Schaun [55] provides an update of the literature on how to perform this verification phase Any type of exercise testing is accepted (e.g., walking, running, or biking) as long as it adapts to the type of activity in which the consumer wearable is intended to be validated In populations unable to perform maximal test, submaximal exercise-based equations might be an alternative to predict *V*O_2max_, since overall these have demonstrated a moderate to strong relationship with maximal tests. However, authors should select the most appropriate equation for their target population [9, 70]	Report whether maximal or submaximal exercise test is being used. In the case of submaximal test, provide a rationale of its implementation and specify the exercise-based equations used In maximal exercise test, report the need for reaching volitional fatigue and indicate the maximum-effort criteria included (at least two criteria) Report the type of exercise testing used as well as its characteristics (e.g., increase in the ramp inclination in treadmill tests or power increase in cycle-ergometer tests)
	Standardized conditions before the reference and index measure	Participants should not consume a significant caloric uptake at least 2 h before the exercise test No caffeine, similar stimulants, or alcohol should be consumed 24 h before the exercise test No intensive sports activities should be performed 48 h before the exercise test Participants should not take any medication that may alter the normal HR response to a maximal exercise The exercise test should begin with at least 2–3 min warm-up	Report the standardized conditions followed by participants Describe the warm-up characteristics
	Wearable device set up	Follow the manufacturer’s instructions for the *V*O_2max_ estimation protocol Provide all the information required by the device, since in some cases this is used to improve the *V*O_2max_ estimation If the device has the option to select a specific exercise mode (i.e., indoor running, cycling, walking, etc.), choose the mode that best reflects the activity that is going to be performed In those wearable devices using GPS data, it is recommended to perform the test outdoor to ensure a proper GPS connection	Report the device model and version Report what demographic details are input into the device per participant for initiation Report what mode (if any) is used during each activity (i.e., indoor running, cycling, walking, etc.) If GPS is used, indicate that the satellite connection was checked before the exercise test
Data processing	Indirect calorimetry processing	If a time average is used to reduce variability in the indirect calorimetry data, typically this should be between 15 and 30 s [26] If a breath average is used, a 15-breath running average is recommended [26] Confirm that the maximum-effort criteria were met when interpreting the *V*O_2max_ values	Report the time-averaged or breath-averaged sampling used Report whether maximal or peak *V*O_2_ is being assessed Detail the data processing conducted in the *V*O_2max_ interpretation
	Time interval between evaluations	If resting conditions are used for wearable *V*O_2max_ estimation, no time interval is needed before the reference *V*O_2max_ test is performed If the wearable test involves exercising, between 24 and 48 h is recommended to ensure an effective muscle recovery. If the maximal test is evaluated first, a time interval between 48 and 72 h is recommended [7]	Report the time interval between both assessments
Statistical analysis	Statistical tests	To assess device accuracy, the following statistical tests should be performed: 1. Bland–Altman with limits of agreement 2. Least product regression of the difference against the means 3. MAPE Subgroup analysis is encouraged if sample size allows. (e.g., sex, age category, ethnicity, BMI)	Include Bland–Altman plots for a visual inspection of the validity results Binary conclusions about the validity of the device should not be made if a formal sample size analysis has not been conducted

*ACSM* American College of Sports Medicine, *BMI* body mass index*, HR* heart rate, *MAPE* mean absolute percentage error, *PPG* photoplethysmography, *VO*_*2max*_ maximal oxygen consumption

**Table 3:** The INTERLIVE checklist to be considered for the validation protocol of wearable to estimate maximal oxygen consumption (*V*O_2max_)

Target population assessment
Age
Children (< 12 years)
Adolescents (12–18 years)
Adults (18–65 years)
Older adults (> 65 years)
Sex (equal sample of males and females)
Sample size Calculated based on previously published or pilot study data OR If previous data is not available, sample of convenience (*n* ≥ 45 participants)
Reference standard
The gold standard is a maximal exercise test in laboratory conditions with indirect calorimetry
Any brand of metabolic cart is accepted and should be calibrated following manufacturer’s instructions
Index device assessment
Consumer wearables placed according to manufacturer’s instructions to be tested in ecological locations Hear rate can be measured with both chest strap or PPG, and it should be reported which of them was used
Testing protocols and conditions
*Reference standard*
To consider at least 2 maximal-effort criteria during the incremental test
A verification phase after the maximal test is recommended to corroborate the *V*O_2max_
Any type of exercise testing is accepted (e.g., walking, running, or biking) as long as it adapts to the type of activity in which the consumer wearable is intended to be validated
Control the standardized conditions before the maximal exercise test
*Consumer wearable*
Follow the manufacturer’s instructions for the *V*O_2max_ estimation protocol
Provide all the setup information required by the devices
If exercise mode is available, choose the one that best reflects the activity to be performed
Ensure an optimal GPS connection when this data is used
Processing
*Reference standard*
If *V*O_2max_ is averaged within a time window, it is recommended to use a 15- to 30-s window
If a breath-by-breath average is used, a 15-breath running average is recommended
Confirm that the maximum-effort criteria were met when interpreting the *V*O_2max_ values
*Time interval between evaluations*
In those wearables using resting conditions, no time interval is needed
In exercise conditions, an interval between 24 and 48 h is recommended
Statistical analysis
Bland–Altman with limits of agreement
Least products regression of the differences against the means
MAPE

See the Table 2 for more detailed information about each item

*INTERLIVE* Towards Intelligent Health and Well-Being Network of Physical Activity Assessment, *MAPE* mean absolute percentage error, *PPG* photoplethysmography

## Discussions, Future Directions, and Statement

In the present article, we combined a systematic review and meta-analysis with an expert statement aiming (1) to provide a summary of the validity of *V*O_2max_ estimations by consumer wearables that use different methods/algorithms and (2) to provide recommendations for future validation studies. Our meta-analysis suggests that consumer wearables using exercise tests provided a more accurate estimation of *V*O_2max_ in comparison to consumer wearables using resting tests. Overall, the wearables using exercise tests to estimate *V*O_2max_ had a systematic error close to zero (− 0.09 ml·kg^−1^·min^−1^) in comparison to maximal graded exercise tests using indirect calorimetry in laboratory conditions. However, the random error observed in both types of methods was still large, i.e., limits of agreements span of ± 15.24 (95% CI − 22.18 to 26.53) and ± 9.83 (95% CI − 16.79 to 16.61) ml·kg^−1^·min^−1^ for the resting and exercise tests, respectively. Consequently, even if this random error was markedly smaller in the exercise-based estimations, it is still a large error when estimating *V*O_2max_ at an individual level.

We are unaware of any well-established and accepted estimation error to strongly indicate when the validity of a wearable is acceptable or not. Our aim here was to inform the public about the observed estimation errors based on existing literature. It is ultimately up to the users to consider whether the error is good enough for their specific purposes. Just to put into context the potential meaningfulness of estimation errors observed in *V*O_2max_, we need to consider that previous meta-analyses have reported that increases in *V*O_2max_ of 1.75–3.5 ml·kg^−1^·min^−1^ are associated with a lower risk of all-cause mortality and incidence of coronary heart disease or cardiovascular disease [5, 64]. Therefore, systematic and random errors in the estimation by wearables beyond the range of 3.5 ml·kg^−1^·min^−1^ will be missing clinically relevant changes. Reliability is also an important concept to understand the quality of the wearables estimates; however, only three of the included studies evaluated it [40, 41, 47]. Overall, good test–retest reliability of wearable *V*O_2max_ has been reported with *r* and intraclass correlation coefficient (ICC) values above 0.90, but further studies using a more recommendable approach (i.e., Bland–Altman limits of agreement) are needed to confirm that wearable *V*O_2max_ is reliable. Given the lack of evidence regarding reliability, caution should be paid when wearables are used for testing individual changes for either research, clinical, or sports purposes. On the other hand, the estimation errors of the exercise-based algorithms at the group level show a high level of accuracy. This fact allows digital phenotyping of cardiorespiratory fitness using wearables at a population level, which opens new opportunities for fitness monitoring at regional, national, or global levels. We cannot determine the number of people for which the exercise-based algorithms are accurate, but considering our results come from 244 participants, we can establish this population cut-off point for now.

In order to better understand the different errors observed in the two types of estimation methods, it is important to discuss how the different brands estimate *V*O_2max_ through different methodologies. Polar devices use resting HR, HRV, gender, age, height, body weight, and self-reported physical activity to estimate *V*O_2max_. The company explains in a white paper that they used data from several validation studies to develop an artificial neural network that calculates *V*O_2max_ through the fitness test [65]. They claim that the mean error of the prediction varies between 8% (3.7 ml·kg^−1^·min^−1^ approximately) and 15% compared with laboratory test. Our results reveal an assumable systematic error of 2.17 ml·kg^−1^·min^−1^, but an overly wide random error span of ± 30.48 ml·kg^−1^·min^−1^. Polar claims the main benefit of the Polar fitness test is that it is “easy, safe and convenient for setting a baseline and tracking relative progress” [57]. We agree that a test in resting conditions is very convenient, feasible, and safe and, therefore, a good solution when more valid methods are not feasible. However, based on the wide random error observed in the meta-analysis, we would not advise users to rely on the estimated *V*O_2max_ from resting conditions, and future efforts to improve this methodology are required.

Fitbit and Garmin use the algorithms developed by Firstbeat Technologies in the *V*O_2max_ estimation [29, 44, 46]. This method uses the following calculation steps [66]: (1) logging of personal information (at least age), (2) an exercise test with the wearable measuring HR and speed, (3) HR data are segmented to different zones and the reliability of these segments is calculated, and (4) the most reliable data segments are used to estimate *V*O_2max_ by using linear or nonlinear dependency between HR and speed data. The white paper published by Firstbeat stated that this estimation had 5% MAPE for running, 8% for cycling, and 6% for walking against indirect calorimetry *V*O_2max_ in laboratory settings [66]. Four studies in this systematic review reported MAPE analyses of Fitbit and Garmin devices in running tests [25, 39, 44, 46], and results were always greater than the 5% reported by Firstbeat, with values ranging from 8 to 10.2%. There are no standard thresholds to determine an optimal MAPE, but previous validity studies of consumer-based wearables considered ≥ 10% as an indicator of inaccuracy, which are values close to those found in the exercise protocols [67]. Although the systematic error we found in the meta-analysis for these wearables using exercise tests is negligible (i.e., 0.09 ml·kg^−1^·min^−1^), the random error span of ± 9.83 ml·kg^−1^·min^−1^ represents a considerable range that may consider its use inappropriate to adequately assess and monitor *V*O_2max_ changes. Nevertheless, this estimation methodology is clearly superior to the resting approach with 2.08 and 10.82 ml·kg^−1^·min^−1^ less systematic and random error, respectively. By removing articles prior to 2017, the resting condition demonstrated an improvement in the accuracy of 0.51 ml·kg^−1^·min^−1^. This analysis supports the notion that new devices and/or algorithms are providing more accurate estimates. Nevertheless, results from this article should encourage developers to opt for exercise methodologies for a more accurate *V*O_2max_ estimation.

This article has detected several weaknesses in the validation process, which highlights the need for further and more rigorous studies. Future validation studies should consider the best-practice recommendations provided in this article by the INTERLIVE consortium in the six main domains. Our review has detected that the validity of wearables has been tested only in healthy and physically active people with a narrow age range (i.e., 25 ± 6 years). A recent systematic review identified several determinants of cardiorespiratory fitness such as sex, age, education, socioeconomic status, ethnicity, body mass index (BMI), body weight, waist circumference, body fat, resting HR, C-reactive protein, smoking, alcohol consumption, and physical activity level [68]. Future validity studies should include participants across the spectrum of some of these influencing factors to determine how the wearable *V*O_2max_ performs in different populations. Moreover, the reference standard and its associated protocol and data processing were, without a doubt, the most critical point in terms of risk of bias in the included studies. Therefore, future studies should improve the indirect calorimetry protocols used according to the current exercise testing guidelines.

Regarding the wearable devices, greater transparency from companies regarding not only the algorithms but also the data used to estimate *V*O_2max_ would be desirable (yet limited by proprietary issues). This would help researchers to better control variables during validation protocols. For instance, if running speed and inclination are used in the estimation, then the quality of GPS signal, track maps, and altimeter sensors should be key components to consider in validation studies. HR seems to provide key data in the *V*O_2max_ estimation, and a great proportion of the consumer wearables in this review included chest strap for the HR measurement instead of PPG. Overall, our results in the meta-analyses demonstrated a greater bias and limit of agreement in those devices using PPG compared to chest strap. This is a somewhat expected finding since the measurement error of the chest strap seems minimal compared to electrocardiogram monitoring [69]. However, since wearing chest straps is uncomfortable for many people and the greater acceptability in the general population of HR monitoring via PPG (usually placed on the wrist, i.e., smartwatches and bracelets), it is important that future validity studies use PPG technology and aim to obtain accurate *V*O_2max_ estimations with it. In a previous INTERLIVE article, we discussed several factors affecting the accuracy of PPG technology, such as skin tone, motion artifacts, contact pressure, and ambient temperature [19]. Recommendations from this article should be considered to ensure best practice in the validity, testing, and reporting of PPG-based HR wearables estimating *V*O_2max_. Lastly, all available literature estimated *V*O_2max_ while running. Thus, future validity studies are needed in other activities, such as cycling or walking, to cover a broader range of activities.

The statistical analysis used in the available validity studies was often inappropriate, and consequently, future protocols should use the statistical approaches considered appropriate in validation studies. We recommend using the Bland–Altman limits of agreement as the main analysis and some percentage error (e.g., MAPE) as complementary and informative information. Overall, the application of the best-practice recommendations from the INTERLIVE consortium would be beneficial for stakeholders by ensuring a more valid and transparent metric derived from their devices as well as for users who would receive more accurate and reliable information about their *V*O_2max_ level and, therefore, their health status.

## Conclusion

This systematic review and meta-analysis from the INTERLIVE consortium summarizes the validity of *V*O_2max_ estimated from consumer wearables and provides best-practice recommendations for future validation protocols. The meta-analysis suggests that the estimation of *V*O_2max_ by wearables that use exercise-based algorithms provides higher accuracy than those based on resting methods. The exercise-based estimation seems to be optimal for application at the population level, yet the estimation error at the individual level and, therefore, use for sport/clinical purposes still needs further improvement. The INTERLIVE network hereby provides best-practice recommendations to be used in future protocols to move towards a more accurate, transparent, and comparable validation of *V*O_2max_ derived from wearables.

## Supplementary Information

Below is the link to the electronic supplementary material.

Supplementary file1 (DOCX 3841 KB)

## Declarations

### Data availability statement

This systematic review has no original data to provide. Most of the data have been reported within the main text or supplementary material. The database used for the meta-analysis and the R script for the Bland–Altman limits of agreement analysis is available upon request to the corresponding authors.
