# Supplementary material for: Validity of Estimating the Maximal Oxygen Consumption by Consumer Wearables: A Systematic Review with Meta-analysis and Expert Statement of the INTERLIVE Network
Source: Sports Med. 2022 Jan 24;52(7):1577–97. doi: 10.1007/s40279-021-01639-y (PMC9213394; doi:10.1007/s40279-021-01639-y)
Supplement: Supplementary file 1 — Supplementary file1 (DOCX 3841 KB) [file 40279_2021_1639_MOESM1_ESM.docx]

# **Validity of estimating the maximal oxygen consumption by consumer wearables: A systematic review with meta-analysis and expert statement of the INTERLIVE network**

**Journal name:** Sports Medicine

Pablo Molina-Garcia ^1,2,^*, Hannah L. Notbohm ^3^, Moritz Schumann ^3,4^, Rob Argent ^5,6,7^, Megan Hetherington-Rauth ^8^, Julie Stang ^9^, Wilhelm Bloch ^3^, Sulin Cheng ^3,4^, Ulf Ekelund ^9^, Luis B Sardinha ^8^, Brian Caulfield ^5,6^, Jan Christian Brønd ^10^, Anders Grøntved ^10^, Francisco B Ortega ^1,11,12^

1. PROFITH (PROmoting FITness and Health through physical activity) Research Group, Department of Physical Education and Sports, Faculty of Sport Sciences, University of Granada, Granada, Spain
2. Biohealth Research Institute, Physical Medicine and Rehabilitation Service, Virgen de las Nieves University Hospital, Jaén Street, s/n, 18013 Granada, Spain
3. Institute of Cardiovascular Research and Sports Medicine, Department of Molecular and Cellular Sports Medicine, German Sport University, Cologne, Germany
4. Exercise Translational Medicine Centre, the Key Laboratory of Systems Biomedicine, Ministry of Education, and Exercise, Health and Technology Centre, Department of Physical Education, Shanghai Jiao Tong University, Shanghai, China
5. Insight Centre for Data Analytics, University College Dublin, Dublin, Ireland.
6. School of Public Health, Physiotherapy and Sport Science, University College Dublin, Ireland
7. School of Pharmacy and Biomolecular Sciences, Royal College of Surgeons in Ireland, Dublin, Ireland
8. Exercise and Health Laboratory, CIPER, Faculdade de Motricidade Humana, Universida de de Lisboa, Lisboa, Portugal
9. Department of Sport Medicine, Norwegian School of Sport Sciences, Oslo, Norway
10. Department of Sports Science and Clinical Biomechanics, Research Unit for Exercise Epidemiology, Centre of Research in Childhood Health, University of Southern Denmark, Odense M, Denmark
11. 11. Faculty of Sport and Health Sciences, University of Jyväskylä, Jyväskylä, Finland
12. Department of Bioscience and Nutrition, Karolinska Institutet, Huddinge, Sweden

***Corresponding authors:**

Pablo Molina-Garcia and Francisco B Ortega, Faculty of Sports Science, University of Granada, Carretera de Alfacar s/n, Granada 18071, Spain. Tel. +34 958 244353. E-mail: [pablomolinag5@gmail.com](mailto:pablomolinag5@gmail.com) and [ortegaf@ugr.es](mailto:ortegaf@ugr.es)

| **Supplementary Material 1.** Search terms used in Scopus, PubMed, and Web of Science databases excluding study design. | | |
| --- | --- | --- |
| **Scopus** | **Web of Science** | **Pubmed** |
| **Index device**  TITLE-ABS-KEY ( wearable* OR smartwatch* OR "smart watch" OR "smart watches" OR watch* OR smartband* OR "smart band" OR "smart bands" OR smartbracelet* OR "smart bracelet" OR "smart bracelet" OR "smart bracelets" OR “tracker” OR “trackers” OR "fitness tracker" OR "fitness trackers" OR “monitor”) | **Index device**  ALL=( wearable* OR smartwatch* OR "smart watch" OR "smart watches" OR watch* OR smartband* OR "smart band" OR "smart bands" OR smartbracelet* OR "smart bracelet" OR "smart bracelet" OR "smart bracelets" OR “tracker” OR “trackers” OR "fitness tracker" OR "fitness trackers" OR “monitor”) | **Index device**  ( wearable* OR smartwatch* OR "smart watch" OR "smart watches" OR watch* OR smartband* OR "smart band" OR "smart bands" OR smartbracelet* OR "smart bracelet" OR "smart bracelet" OR "smart bracelets" OR “tracker” OR “trackers” OR "fitness tracker" OR "fitness trackers" OR “monitor”) |
| **Outcome**  TITLE-ABS-KEY ("VO2max" OR "VO2 max" OR "VO2maximum" OR "VO2 maximum" OR "VO2peak" OR "VO2 peak" OR "oxygen uptake" OR "O2 uptake" OR "oxygen consumption" OR "O2 consumption" OR "aerobic capacity" OR “cardiorespiratory”) | **Outcome**  ALL=("VO2max" OR "VO2 max" OR "VO2maximum" OR "VO2 maximum" OR "VO2peak" OR "VO2 peak" OR "oxygen uptake" OR "O2 uptake" OR "oxygen consumption" OR "O2 consumption" OR "aerobic capacity" OR “cardiorespiratory”) | **Outcome**  ("VO2max" OR "VO2 max" OR "VO2maximum" OR "VO2 maximum" OR "VO2peak" OR "VO2 peak" OR "oxygen uptake" OR "O2 uptake" OR "oxygen consumption" OR "O2 consumption" OR "aerobic capacity" OR “cardiorespiratory”) |
| **Study Design**  TITLE-ABS-KEY (Valid* OR accura*) | **Study Design**  ALL=(Valid* OR accura*) | **Study Design**  (Valid* OR accura*) |
|  |  |  |
| **N studies included:** 706 | **N studies included:** 525 | **N studies included:** 433 |

| **Supplementary Material 2.** Criteria for the risk of bias assessment | |
| --- | --- |
| Criteria items | Number and percentage of studies meeting criterion out of 14 studies included. N (%) |
| **DOMAIN 1: Participants** |  |
| 1. Were participants selected appropriately to represent the desired target population defined by study authors? | 11 (78.6) |
| 1. Did all or nearly all participants sampled for the study, contribute with data to be included in the analysis of criterion validity? | 11 (78.6) |
|  |  |
| **DOMAIN 2: Index Measure** |  |
| 1. Was the wearable device administered during sampling of data according to manufacturer’s instructions? | 10 (71.4) |
|  |  |
| **DOMAIN 3: Reference Standard** |  |
| 1. Was the criterion measure instrument to assess VO_2_ appropriate and administered appropriately during the test? | 10 (71.4) |
| 1. Was the test protocol (including criteria for a valid test) for VO_2_-max assessment appropriate? | 9 (64.3) |
| 1. Did the professional(s) administer the VO_2_-max test (reference) without knowledge of values from the wearable device? | 4 (28.6) |
| 1. Was the time interval between reference and index measurements appropriate? | 9 (64.3) |
|  |  |
| **DOMAIN 4: Statistical Analysis** |  |
| 1. Was the statistical approach to estimate agreement appropriate? | 8 (57.1) |
|  |  |

**Supplementary Material 3.** Risk of bias assessment of each article using the COSMIN tool and the Risk of Bias 2 (RoB 2) criteria.

| **Supplementary Material 4.** Leave-one-out method to test the robustness of the meta-analysis. | | | |
| --- | --- | --- | --- |
| **Methodology** | **Study** | **Bias (95% IC)** | **Z (p)** |
| **Resting test** | **All Studies included** | **2.17 [0.28, 4.07]** | **2.25 (0.020)** |
|  | Cooper et al. (2019) | 2.50 [0.48, 4.52] | 2.42 (0.020) |
|  | Crouter et al. (2004). Men | 2.42 [0.42, 4.42] | 2.38 (0.020) |
|  | Crouter et al. (2004). Women | 1.49 [-0.13, 3.31] | 1.80 (0.070)* |
|  | Esco et al. (2011) | 2.66 [0.81, 4.51] | 2.82 (0.005) |
|  | Esco et al. (2014) | 1.96 [-0.16, 4.09] | 1.81 (0.070)* |
|  | Kraft and Dow (2019) | 2.10 [0.03, 4.16] | 1.99 (0.050) |
|  | Lowe et al. (2010) | 2.14 [-0.12, 4.39] | 1.86 (0.060)* |
|  | Passler et al. (2019) | 2.09 [-0.01, 4.19] | 1.95 (0.050)* |
|  | Snyder et al. (2019). Men | 1.92 [-0.11, 3.94] | 1.86 (0.060)* |
|  | Snyder et al. (2019). Women | 2.55 [0.58, 4.52] | 2.54 (0.010) |
|  |  |  |  |
| **Exercise test** | **All Studies included** | **-0.09 [-1.66, 1.48]** | **0.11 (0.910)** |
|  | Anderson et al. (2019) | 0.17 [-1.54, 1.88] | 0.20 (0.850) |
|  | Carrier et al. (2020) | -0.38 [-2.00, 1.25] | 0.46 (0.650) |
|  | Freeberg et al. (2019) | -0.45 [-2.02, 1.12] | 0.57 (0.570) |
|  | Klepin et al. (2019) | -0.06 [-1.86, 1.74] | 0.07 (0.950) |
|  | Kraft and Dow (2019) | -0.14 [-1.84, 1.56] | 0.16 (0.870) |
|  | Passler et al. (2019) | 0.17 [-1.55, 1.89] | 0.19 (0.850) |
|  | Snyder et al. (2019). Men | -0.28 [-1.98, 1.42] | 0.33 (0.740) |
|  | Snyder et al. (2019). Women | -0.19 [-1.96, 1.58] | 0.21 (0.830) |
|  | Wagner et al. (2020) | 0.38 [-0.86, 1.61] | 0.60 (0.550) |
| * : When leaving out this study, the overall effect changes significantly in comparisson with the overall effect including all the studies. | | | |

**Supplementary Material 5.** Funnel plots and Egger’s tests to assess the publication bias in both the orthostatic-test and exercise-test studies.

**Supplementary Material 6.** Pooled bias and standard error (SE) for wearables VO_2_max using photoplethysmography (PPG) for heart rate recording (panel A) versus wearables using chest strap (panel B) in the resting conditions. A negative bias represents an underestimation and a positive an overestimation of the VO_2_max by the wearables in comparison to the reference standard.

**Supplementary Material 7.** Pooled bias and standard error (SE) for wearables VO_2_max using photoplethysmography (PPG) for heart rate recording (panel A) versus wearables suing chest strap (panel B) in the exercise tests. A negative bias represents an underestimation and a positive an overestimation of the VO_2_max by the wearables in comparison to the reference standard.

| **Supplementary Material 8.** Consumer wearable devices identified in the market providing VO_2_max estimations and their main characteristics. | | | | | | |
| --- | --- | --- | --- | --- | --- | --- |
| **Manufactor** | **Devices** | **Sensors used** | **Additional measures included** | **Constraints** | **Protocol** | **Source** |
| Apple | Watch 6,  WatchOS 7 | HR | Age, gender, weight |  | 6-minute walk test 20 min workout | [Link](https://www.apple.com/healthcare/docs/site/Using_Apple_Watch_to_Estimate_Cardio_Fitness_with_VO2_max.pdf) |
| Garmin | All Forerunner All Fenix All Instinct | HR and GPS | Age, gender, weight | Need GPS for walking and running Cycling needs power meter | Running 10 minutes (15 minutes with vivosport) Cycling: Minimum of 20 minutes and HR must be at least 70% of HR_max_ Walking: Minimum 10 minutes and HR must be at least 70% of HR_max_ Cycling – Minimum of 20 minutes | [Link](https://support.garmin.com/en-US/?faq=lWqSVlq3w76z5WoihLy5f8) |
| TomTom | Spark Cadio | HR and GPS | Age, gender, weight | Steady HR | min 15 minutes of running, trail running or treadmill | [Link](https://help.tomtom.com/hc/en-us/articles/360013899420-VO2max-and-Fitness-Age-calculation) |
| Polar | V800 | HRV | Age, gender, weight |  | During resting conditions | [Link](https://support.polar.com/e_manuals/V800/Polar_V800_user_manual_English/Content/Fitness_Test.htm) |
| Polar | Polar Vantage V2 Polar Unite Polar Grit X Polar H10 and OH1 (chest strap) | HR and GPS | Age, gender, weight |  | 5 min fitness test | [Link](https://www.polar.com/blog/lets-talk-polar-polar-fitness-test/) |
| Huawei | GT2 and GT3 | HR and GPS | Age |  | Running of at least 2.4 km within 20 min | [Link](https://consumer.huawei.com/us/wearables/watch-gt/) |
| Suunto | Suunto 3, 5, 7 and 9 | HR and GPS | Age, gender and weight |  | At least 15 minutes of outdoor walking or running | [Link](https://www.suunto.com/da-dk/Support/Product-support/suunto_5/suunto_5/funktioner/fitnessniveau/) |
| Withings | Steel HR sport | HR and GPS | Age, gender, weight |  |  | [Link](https://support.withings.com/hc/en-us/articles/360009326614-Steel-HR-Sport-What-is-VO2-max-and-does-the-watch-measure-it-) |
| Coros | APEX | HR and GPS |  |  |  | [Link](https://coros.com/apex.php) |
|  |  |  |  |  |  |  |

Note: The brands and models might be subject to constant changes and, therefore, this table should be used as a set of examples identified in the market at the moment of doing this review article, but there might be other wearables estimating VO_2_max and that were missed during the search
